# Homologous recombination deficiency (HRD) testing landscape: clinical applications and technical validation for routine diagnostics

**DOI:** 10.1186/s40364-025-00740-y

**Published:** 2025-02-21

**Authors:** Andréa Witz, Julie Dardare, Margaux Betz, Cassandra Michel, Marie Husson, Pauline Gilson, Jean-Louis Merlin, Alexandre Harlé

**Affiliations:** Département de Biopathologie, Institut de Cancérologie de Lorraine, CNRS UMR 7039 CRAN - Université de Lorraine, Vandoeuvre-lès-Nancy, France

**Keywords:** Homologous recombination deficiency, HRD status, PARP inhibitors, Genomic scars analysis, *BRCA* mutations testing

## Abstract

The use of poly(ADP-ribose) polymerase inhibitors (PARPi) revolutionized the treatment of *BRCA*-mutated cancers. Identifying patients exhibiting homologous recombination deficiency (HRD) has been proved useful to predict PARPi efficacy. However, obtaining HRD status remains an arduous task due to its evolution over the time. This causes HRD status to become obsolete when obtained from genomic scars, rendering PARPi ineffective for these patients. Only two HRD tests are currently FDA-approved, both based on genomic scars detection and *BRCA* mutations testing. Nevertheless, new technologies for obtaining an increasingly reliable HRD status continue to evolve. Application of these tests in clinical practice is an additional challenge due to the need for lower costs and shorter time to results delay.

In this review, we describe the currently available methods for HRD testing, including the methodologies and corresponding tests for assessing HRD status, and discuss the clinical routine application of these tests and their technical validation.

## Background

Genomic integrity of cells is continually challenged throughout their lifespan by endogenous and exogenous events. Following genomic alterations, cells can activate different mechanisms to repair the resulting damages and preserve their genomic integrity. Failure to achieve these mechanisms, errors during lesion repair or occurring spontaneously can lead to genomic instability, which is one of the hallmarks of cancer [1]. Indeed, accumulation of these genomic alterations results in cancer by shortening the cell cycle, giving tumor cells a survival advantage [2]. Targeting tumor cells while sparing healthy cells is one of the major challenges in cancer therapy. The characterization of the genomic instability of tumors is thus an important avenue to exploit for the development of anticancer drugs.

**Fig. 1:**
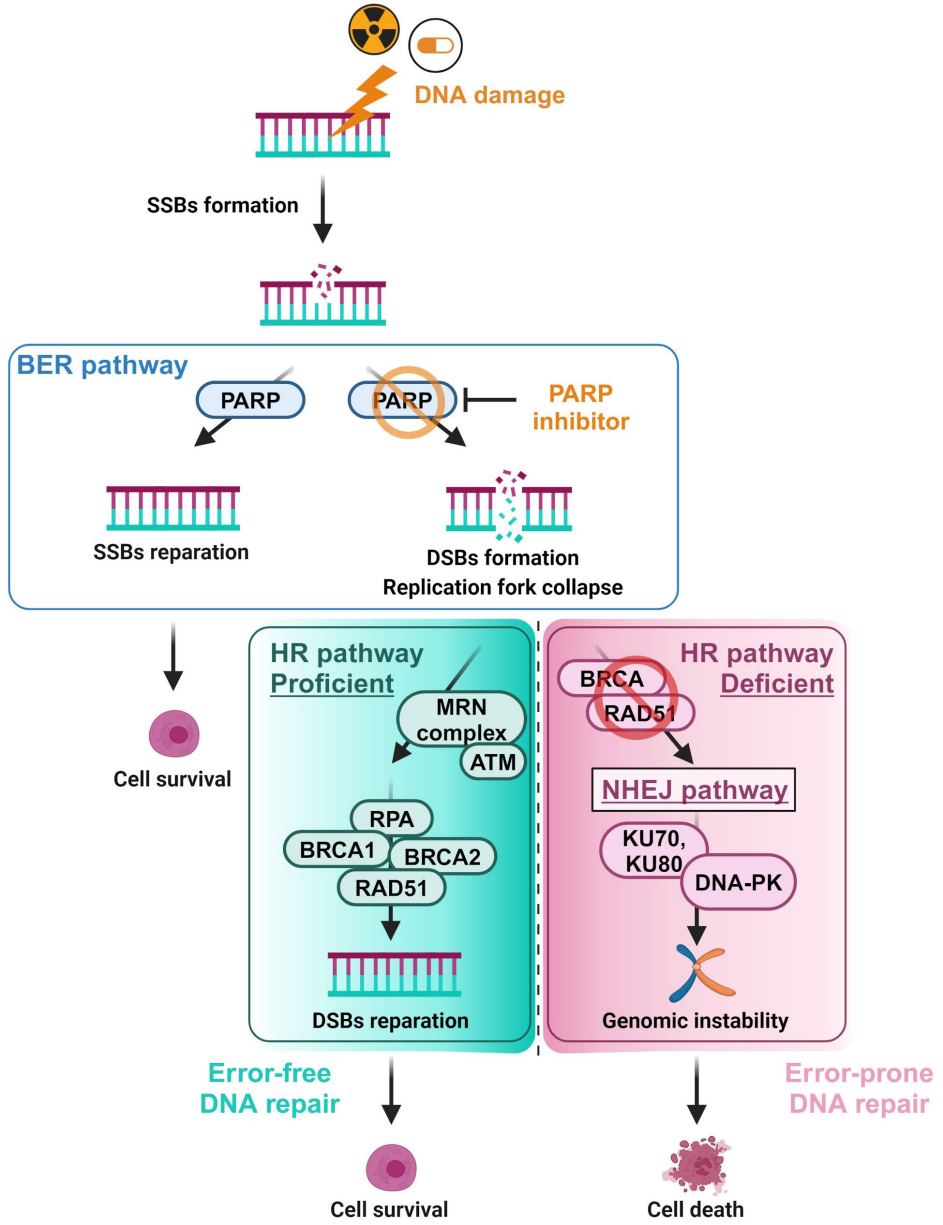
Poly (ADP-ribose) polymerase (PARP) inhibitors mechanism of action in homologous recombination (HR) proficient and deficient cells. When the DNA is damaged, leading to the formation of single-strand breaks (SSBs), the base excision repair (BER) pathway repairs these SSBs, mostly through the involvement of PARP enzymes. If PARP enzymes are trapped or inhibited by PARP inhibitors, SSBs are not repaired, resulting in the formation of double-strand breaks (DSBs) in DNA. These DSBs are preferentially repaired *via* the error-free DNA repair pathway, i.e. the HR repair pathway, which utilises the MRE11-RAD50-NBS1 (MRN) complex to recognize DSBs and BRCA or RAD51 proteins to repair the DNA breaks, thereby allowing the cell to survive. However, this repair pathway can be deficient, e.g. due to pathogenic mutations of *BRCA genes*, forcing the cell to use the more error-prone non-homologous end joining (NHEJ) DNA repair pathway. This low-fidelity repair pathway leads to an accumulation of a genome instability and finally to cell death. *Created using Biorender (*https://www.biorender.com/)

In the 2000s, a new class of anticancer drugs specifically targeting a tumor cell population emerged: the poly (ADP-ribose) polymerase (PARP) inhibitors (PARPi) that target cells with deficient BRCA1 or BRCA2 proteins [3]. The antitumor activity of PARPi is based on synthetic lethality. This concept involves the loss-of-function of two interdependent mechanisms or pathways to induce cytotoxicity, whereas the inactivation of only one has no effect on cell viability. PARP proteins are involved in single-strand breaks (SSBs) repair by base excision repair (BER) pathway and autoPARylation. In presence of PARPi, PARP proteins are inhibited and/or trapped by PARPi. SSBs can therefore no longer be repaired by trapped PARP proteins, resulting in double-strand breaks (DSBs) (Fig. 1) [4]. In normal cells, an alternative DNA repair pathway is activated to repair DSBs, named homologous recombination (HR) repair (HRR) pathway mediated by BRCA proteins. In tumor cells with BRCA1/2 loss, the HRR pathway is deficient (HR deficiency (HRD)), leading to an accumulation of unrepaired DSBs. Tumor cell death is generated by synthetic lethality *via* PARPi in cells with HRD. Pathogenic mutations in *BRCA1* and *BRCA2* genes are thus predictive biomarkers for PARPi sensitivity in breast, ovarian, pancreatic, and prostate cancers, and are approved as companion test for PARPi indications [3]. Germline mutations of *BRCA1/2* were found in around 7% of breast [5] and pancreatic cancers [6], and in 15% of ovarian cancers [7], but only in 1.5% of prostate cancers [8]. Therefore, studies on the sensitivity of PARPi in patients with an HRD-like phenotype independent of *BRCA* mutations have been performed over the past two decades, resulting in the approval of the HRD status as a companion test for the PARPi. This is the concept of “BRCAness”, which describes cellular characteristics that phenocopy those of BRCA-deficient cells. Patients with a BRCAness phenotype are eligible for PARPi therapy in the same way as patients with a *BRCA* mutation [3]. Assessment of HRD is clinically relevant because HRD is an important predictive biomarker, but it remains an important challenge due to the complexity of HRD mechanism.

This review provides an overview of methods for HRD analysis and available technologies for HRD testing, focusing on their advantages and limitations in routine diagnostics. Additionally, we discuss the technical and clinical validations of these HRD tests.

## HRD analysis

### Causes of HRD

DNA DSBs repair by HR is a multistep process, involving directly or indirectly numerous genes, the two main actors being *BRCA1* and *BRCA2 genes*. Briefly, DSBs are recognized by the MRN complex including MRE11, RAD50 and NBS1, following by ATM activation. BRCA1, CHEK1 and CHEK2 are next recruited at break site and single-strand ends will be created. The lesion is stabilized by replication proteins A (RPA) leading to ATR activation. After PALB2 and BRCA2 recruitment, RPA are replaced by RAD51 proteins, allowing DNA synthesis using homologous DNA as a template, and finally DSBs repair which involved other HR subpathways [9]. Cells capable of repairing DNA DSBs *via* the HRR pathway are known as HR proficient (HRP). In contrast, DNA DSBs repair by the HRR pathway in cells with HRD are ineffective (Fig. 1).

**Fig. 2:**
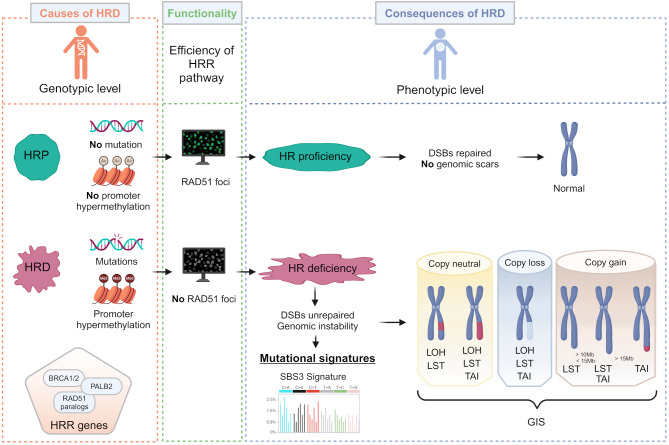
Detection and estimation of homologous recombination (HR) deficiency (HRD) or proficiency (HRP) status through causes and consequences of HRD and HR efficiency. The causes of HRD are observed at genotypic and epigenetic levels and are obtained through the analysis of HR genes, including *BRCA1*, *BRCA2*, *PALB2* or *RAD51* paralogs. In this way, gene mutational status and promoter methylation are studied. HR efficiency can be estimated through a functional test, which allows the identification of the formation of RAD51 foci. The consequences of HRD are observed at the phenotypic level and are determined through the analysis of mutational signatures and genomic scars. With regard to mutational signatures, the single base signature 3 (SBS3) is associated with HRD. With respect to genomic scars, three signatures of chromosomal abnormalities are related to HRD: the loss of heterozygosity (LOH), large-scale transitions (LSTs), and telomeric allelic imbalance (TAI). The unweighted sum of the three signatures is referred to as the genomic instability score (GIS). HRR, homologous recombination repair; DSBs, double-strand breaks. *Created using Biorender (*https://www.biorender.com/)

HRD can be the consequence of different events: somatic or germline deleterious or suspected deleterious mutations in genes directly or indirectly involved in HRR pathway (*e.g. BRCA1*, *BRCA2*, *PALB2* or *RAD51* paralogs) and *BRCA1* [10, 11] or *RAD51C* promoter hypermethylation [12, 13]. The most studied causes of HRD are *BRCA* mutations but an HRD-like phenotype is often observed in patients without *BRCA* mutation referred as BRCAness. The identification of loss-of-function mutation in HR-related genes can give an estimation of HRD status at the genotypic level and inform about causes of HRD. The list of genes implicated in the HR pathway and its associated pathways can be found in the Kyoto Encyclopedia of Genes and Genomes (KEGG) pathway database (Fig. 2) [14].

### Consequences of HRD

Genomic instability is the consequence of defects in the DNA repair machinery, because of accumulation of aberrant DNA alterations and mostly due to unrepaired DSBs. HR and non-homologous end joining (NHEJ) are the two major DSBs repair pathways. HR is more accurate than NHEJ which is an error-prone repair pathway leading to genomic instability [15]. In normal cells, both of those DSBs repair pathways are used in a balanced way, which allows the maintenance of genomic integrity [16]. However, a deficiency in HRR pathway forces cells to use the alternative NHEJ pathway, that induces chromosomal rearrangements, apoptosis, and genomic instability [15]. Consequences of HRD can provide insight into HRD at the phenotypic level (Fig. 2). Because of the absence of a functional HR, HRD can lead to copy number variations (CNVs)), insertions/deletions (indels), mutations, and structural chromosomal abnormalities or rearrangements. Large-scale genomic defects caused by defective DNA repair, also called “genomic scars”, can be divided into three signatures of HRD-related genomic alterations [17].

The loss of heterozygosity (LOH) is one of the three genomic signatures that can be associated with HRD. This genomic aberration is defined as the irreversible loss of one of the parental alleles at a specific chromosomic locus with the consequent absence of one or more tumor suppressor gene(s). LOH can result from deletion of one allele, called “copy-loss LOH”, or from deletion followed by a duplication of the remaining allele, the “copy-neutral LOH” [18]. The length of region of LOH and percentage of genomic LOH (gLOH) can influence the HRD status. Indeed, Abkevich et al. demonstrated that a LOH size > 15 Mb but less than the complete chromosome is significantly correlated with deficiency in HR genes (*BRCA1*, *BRCA2* or *RAD51C*) [19]. Swisher et al. showed that a percentage of gLOH ≥ 14% (LOH_high_) allowed to classify tumors as HRD [20].

The telomeric allelic imbalance (TAI) is another genomic signature linked to the HRD phenotype. TAI is described as the number of subtelomeric regions with allelic imbalance (copy loss or copy gain) but without crossing the centromere. The number of TAI (N_tAI_) is correlated with impaired DSB repair and therefore, HRD. It is known that BRCA loss is often linked to this type of chromosomal aberration, leading to cisplatin sensitivity. According to the study of Birkbak et al., a N_tAI_ ≥22 is also synonymous with cisplatin sensitivity in the context of wild-type (wt) *BRCA* tumors. TAI chromosomal breakpoints are enriched with 25 Kb CNVs and are non-random, suggesting that TAIs formation is the result of stalled replication forks, increased replication stress, or another CNV-associated mechanisms [21].

The large-scale transition (LST) is the third genomic alteration, which lead to genomic scar. LST consists in a chromosomal break between adjacent regions larger than 10 Mb. LSTs can be deletions, inversions, or translocations, but the majority of LSTs is chromosomal translocations with high GC-content. To be considered as HRD, tumors must exhibit 15 or more LSTs if near-diploid, or ≥ 20 LSTs if near-tetraploid. Moreover, tumors can be LST_high_ even if no *BRCA1*, *BRCA2* or *RAD51C* mutations are found [22].

LOH, TAI and LST are independently associated with HRD and *BRCA1/2* dysfunction (due to promoter hypermethylation, and germline or somatic alterations). Although these three scores are correlated with each other, their combination appears to be more effective and robust in detecting HRD tumors from HRP tumors than solely relying on one of the three parameters. Indeed, the unweighted sum of LOH, TAI and LST, called the “HRD score” or “Genomic Instability Score (GIS)” or “HRD-sum”, showed better performance than each signature used alone. This composite score is well suited to differentiate HRD from HRP tumors in breast [23], ovarian [24] and prostate cancers [25], and to predict the platinum-based chemotherapies response.

These three types of genomic scars are an accurate representation of genomic instability and can provide insight into the different DNA repair pathways that occurred over time. However, genomic scars are permanent, in contrast to HRD status, which is more of a dynamic event. Reversion mutations in HR-genes occurring after solid biopsy can restore the HRR pathway and consequently render the patient resistant to PARPi. Thus, the GIS may not properly represent HRD status at the time of treatment [26].

In addition to large genomic rearrangements, the number and types of somatic mutations can also be informative on HRD status. Indeed, recent studies demonstrated that different mutational signatures are associated with specific mutational processes such as aging, action of exogenic carcinogens (e.g. ultraviolet light), defects in DNA repair pathways (HR or mismatch repair (MMR) pathways), and over-activation of enzymes (e.g. APOBEC enzymes). These mutational signatures are distributed in six groups according to the Catalogue Of Somatic Mutations In Cancer (COSMIC) version 3.4: copy number (CN), doublet-base substitution (DBS), small indel (ID), single-base substitution (SBS), structural variation (SV), and RNA-SBS [18, 27, 28]. Among these, six mutational signatures were found to be correlated with HRD. The SBS Signature 3 (SBS3) is the most studied signature. Also known as “COSMIC Signature 3 (Sig3)” or “BRCA signature”, SBS3 consists in a pattern of 96 possible SBS types, enriched in patients with *BRCA1/2*, *PALB2* or *RAD51* mutations [29, 30]. Another SBS signature, SBS8, characterized by C > A, C > T and T > A substitutions, is probably associated with HRD and found in patients with *BRCA1* or *BRCA2* mutations [31]. Moreover, indels signatures ID6 and ID8 are elevated in *BRCA* mutated patients. These microhomology signatures are composed of ≥ 5 bp deletions. Although ID6 is correlated to the SBS3 signature and thus HRD, ID8 is not and refers rather to DNA DSBs repair *via* the NHEJ pathway [28]. In addition to the ID6 signature, the SBS3 signature is also linked with DBS13, which is a signature composed of TC > NN dinucleotide mutations [32], and with CN17. This signature of HRD is characterized by LOH segments with copy number of 2–4 and heterozygous segments with copy number of 3–8, the size of each segment being between 1 and 40 Mb. CN17 is particularly found in patients with bi-allelic loss of HR genes such as *BRCA1*, *BRCA2*, and *PALB2* [33]. Finally, *BRCA1* mutated patients can be enriched in SV3 (known as “Rearrangement signature (RS) 3” in the latest version). This signature consists of tandem duplications of 1-100 kb [31].

### HRD estimation

Estimation of HRD status by functional assays provides the most accurate evidence of a defect in the HR pathway in real-time (Fig. 2). Functional assays measure the DNA repair capacity of cells. The RAD51 Foci Formation assay, introduced by Graeser et al. in 2010, allow to count subnuclear foci of RAD51 created after DNA damage during the S/G2 cell cycle phase [34]. RAD51 protein is recruited by the BRCA1/PALB2/BRCA2 complex at DSBs sites [35]. RAD51 foci are present to the site of DSBs in HRR and reflect the activation of the HRR machinery. Hence, the formation of RAD51 foci is associated with HRR proficiency. HRD status obtained from the RAD51 foci formation assay is correlated with PARPi responses and *BRCA1/2* defects [36], such as in the functional RECAP test [37]. γH2AX foci and 53BP1 foci are also informative regarding DSBs [38]. Nevertheless, clinical routine use of the RAD51 foci formation assay is uncertain. Indeed, this assay remains an experimental approach due to technical discrepancies, such as the methods of detection (immunofluorescence or immunohistochemistry) or the determination of foci (number of RAD51 foci or percentage of foci) [36]. Although Castroviejo-Bermejo et al. showed the routine feasibility of the assay with formalin-fixed paraffin embedded (FFPE) samples [39], no commercial tests are available and the technique was mainly used in clinical trials with an ex-vivo induction of DNA damage [36, 40]. Moreover, the determination of HRD status *via* the RAD51 foci formation assay is impracticable for tumors with a low proliferation rate [36], leading to a high failure rate [41]. To note, the formation of RAD51 foci can be preserved while tumor is HRD through *e.g. ATM* alterations [36].

### HRD detection

Detection of HRD status through genomic features is another way of predicting response to PARPi. Various technologies can be used to detect mutations in HR genes (HRD causes, at genotypic level) or to obtain HRD score (HRD consequences, at phenotypic level) (Fig. 2). Samples analyzed for this purpose can be obtained from a tumor tissue or liquid biopsy. Although tissue biopsy remains the gold standard in clinical practice, liquid biopsy—particularly circulating tumor DNA (ctDNA)—is gaining increasing interest due to its minimally invasive nature, ease of collection, high sampling frequency, and ability to assess intratumor heterogeneity from a single sample [42]. It is noteworthy that the use of ctDNA for HRD testing is increasingly evaluated [43–45]. However, the risk of false-negative results due to the low level of ctDNA should be considered, as well as the risk of false-positive results due to the clonal hematopoiesis of indeterminate potential (CHIP) [46]. The utilization of FFPE or fresh frozen tissue samples for HRD status detection can also be limited by the low tumor content or the poor quality of the samples. In contrast, the clinical utility and validity of these types of sampling have been well-demonstrated, despite the inability to rebiopsy the patient which precludes the possibility of monitoring changes in HRD status over time [47]. For assessing HRD status, Next-Generation Sequencing (NGS) and Single Nucleotide Polymorphism (SNP) arrays are the two main methods.

### Determination of HRD status using single nucleotide polymorphism (SNP) array data

Analysis of chromosomal abnormalities (LOH, TAI, LST, and copy number alterations (CNAs)) was initially performed using FFPE and SNP arrays, followed by an analysis of SNP array data by an algorithm. OncoScan™ provided by ThermoFisher, Infinium CytoSNP-850 K provided by Illumina [48], and Affymetrix^®^ SNP 6.0 provided by Affymetrix [49], are the most used SNP array-based technologies for the assessment of HRD Score, in addition to custom SNP arrays. SNP arrays allow whole genome (WG) CN analysis using probes labelled on both alleles of a SNP. Nowadays, nearly one million SNPs can be genotyped on one chip [50]. The ABSOLUTE [51], GISTIC [52], OncoSNP [53] and PennCNV [54] software, as well as the Allele-specific CN Estimation (ACNE) [55], ASCAT [56] and GenoCN [57] R packages, are commonly used to interpret SNP-arrays data (genome-wide DNA CN). Efficiency of these algorithms depends on CNA burden and tumor purity [58]. The computation of the LOH, LST and TAI signatures to obtain the HRD score can be performed with the R packages OncoscanR or RediScore v2.0 [59]. The SNP array method offers the advantage of being low cost and provides easy to use data analysis. However, only known SNP locations are genotyped, and prior genomic information are required.

### Determination of HRD status using next generation sequencing (NGS) data

**Fig. 3:**
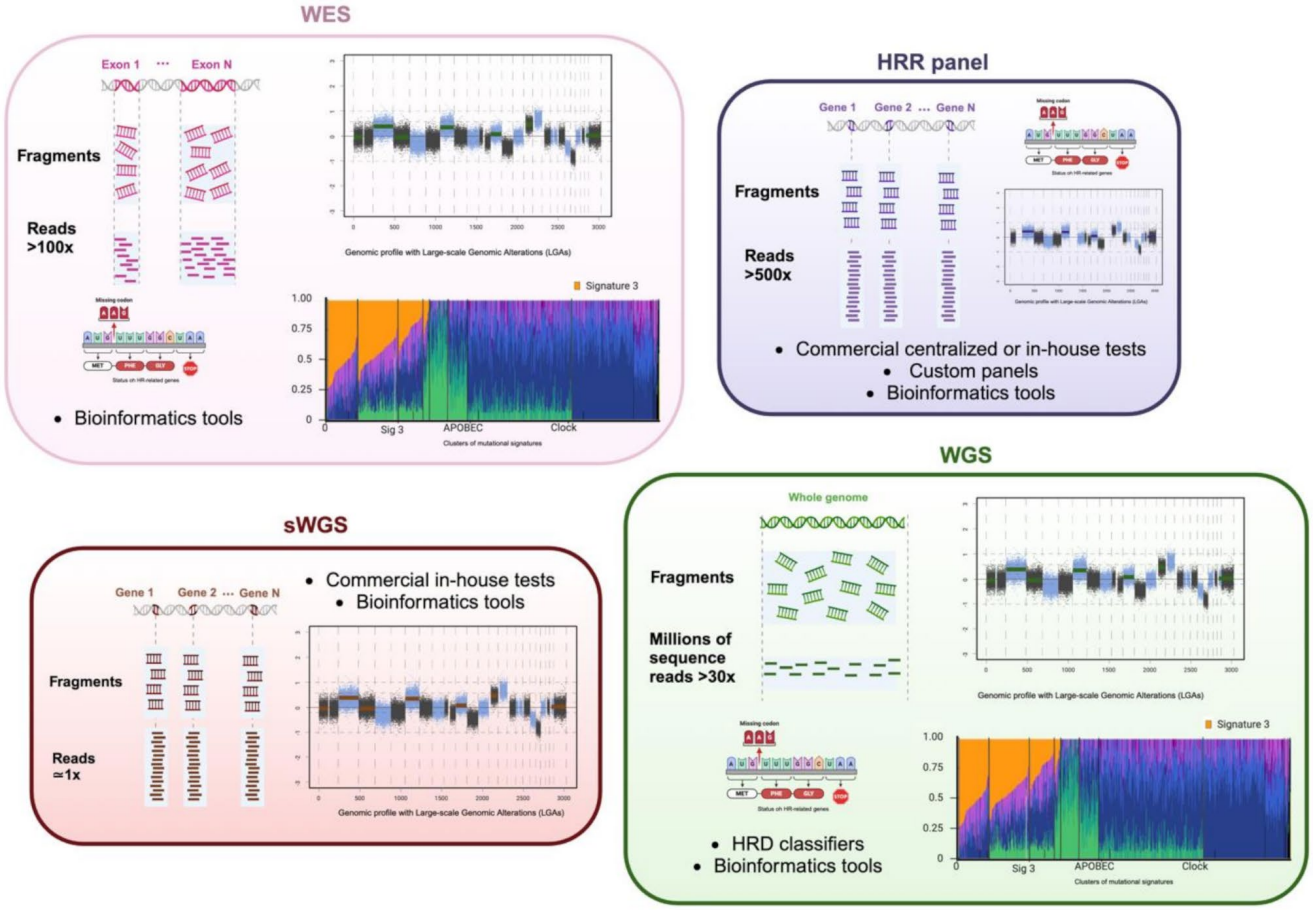
Illustration of methods and corresponding tests for assessing homologous recombination deficiency (HRD) status using next-generation sequencing (NGS) data. The causes of HRD can be assessed by a targeted approach using specific panels of HR-related genes, whole-exome sequencing (WES), or whole-genome sequencing (WGS). These approaches can also identify the consequences of HRD too, in the same way as the shallow WGS (sWGS). HRR, homologous recombination repair. *Created using Biorender (*https://www.biorender.com/)

Although SNP array technology is less expensive than NGS method, resolution of NGS data is higher than that of SNP arrays, and NGS offers better genomic characterization of tumors [60]. As a result, HRD scores based on SNP-arrays have progressively given way to NGS-based HRD scores. There is a good correlation between both HRD scores, validating the use of NGS to obtain the HRD score [61] and also the percentage of gLOH [62]. NGS data are obtained by targeted sequencing panels, shallow WGS (sWGS), whole exome sequencing (WES), or whole genome sequencing (WGS) (Fig. 3).

#### NGS data obtained by specific panels

Targeted gene panel is the least expensive sequencing technique. In addition to being cost-effective, specific panels present the advantage of being faster and easier to implement in clinical practice than other sequencing techniques. Genes known to cause HRD are targeted, and the panel size is ranged between 2 and 700 genes. Hybrid capture-based technology is preferred to amplicon-based one because the detection of large deletions and insertions is required to determine HRD status [63]. Indeed, the hybrid capture-based approach ensures uniform coverage while targeting a large gene region [42], thereby reducing the probability of false positive or negative SNVs, in comparison with amplicon method. Although amplicon-based methods require low-input DNA and less time to prepare the libraries [64], the risk of misdiagnosis by allele dropout and drop-in is higher with this PCR-based method, especially when SNVs are located in the forward and/or reverse primer binding sites [42, 65]. Consequently, hybrid capture emerges as ideal method for clinical applications, ensuring the delivery of precise and reliable results. Two types of targeted panels are commonly used: off-the-shelf panels, which are pre-designed and commercially available, and custom panels, which are rather designed for specific needs.

Commercial in vitro diagnostic tests can be performed in either centralized or decentralized laboratories. Those performed by centralized laboratories have the advantage of not requiring handling. Only block or unstained slides of FFPE tissue are needed and shipped to centralized laboratories. Among them, two tests are approved by the US Food and Drug Administration (FDA) for determination of the HR status. They are referred as companion diagnostics (CDx) and are supplied by only two laboratories: Myriad Genetics and Foundation Medicine. These tests both detect genomic scars but are not interchangeable.

The MyChoice^®^ CDx test, provided by Myriad Genetics, is the first that has been recommended by the American Society of Clinical Oncology (ASCO) and approved by the FDA as HRD companion diagnostic for PARPi treatment in patients with ovarian and prostate cancers. This test is based on the NGS technology using DNA isolated from FFPE samples. Depending on the quality of FFPE samples, MyChoice^®^ CDx consists in the determination of the unweighted sum of LOH, LST and TAI (GIS), and in detection of single nucleotide variations (SNVs), indels and large rearrangements in *BRCA1/2* genes but does not include the determination of whole gene duplications and deletions [23, 66]. Therefore, the test provides information about the consequences of HRD but also about the causes of HRR impairment through *BRCA* genes mutational status. The MyChoice^®^ CDx Plus test includes 13 genes in addition to *BRCA1* and *BRCA2* (*ATM*, *BARD1*, *BRIP1*, *CDK12*, *CHEK1*, *CHEK2*, *FANCL*, *PALB2*, *PPP2R2A*, *RAD51B*, *RAD51C*, *RAD51D*, and *RAD54L*) but the results obtained for these genes are only indicative and are not used for diagnosis [67]. MyChoice^®^ CDx gives a score with a continuous value between 0 and 100 [23]. The threshold for a positive GIS is set at 42 for olaparib (*plus* bevacizumab) as maintenance treatment in advanced ovarian cancers [68] and for niraparib in relapsed ovarian cancers [69]. All tumors with a score below 42 are considered as HRP, and tumors with a score of 42 or upper and/or a pathogenic or a likely-pathogenic mutation in *BRCA1/2* genes are HRD [70]. Based on the results of the VELIA trial (NCT02470585), HRD score ≥ 33 would be more appropriate to identify HRD patients for veliparib indication in ovarian cancers [71].

BRACAnalysis CDx^®^ is another test developed by Myriad Genetics and approved by the FDA as a companion diagnostic for treatment with PARPi in breast, ovarian, pancreatic, and prostate cancers. However, the HRD status cannot be achieved with the BRACAnalysis CDx^®^ test. Indeed, only germline *BRCA1/2* mutations are detected. This germline in vitro test is performed at Myriad Genetics Laboratories, requires whole blood collected in ethylenediamine tetraacetic acid (EDTA) tubes, and consists in the analysis of SNVs and indels by Sanger Sequencing, and the detection of large deletions and duplications of *BRCA1* and *BRCA2* genes by multiplex PCR [72]. Patients with HER2-negative locally advanced or metastatic breast cancer are eligible for treatment with olaparib and talazoparib if a deleterious or suspected deleterious germline *BRCA1/2* mutation is detected by the BRACAnalysis CDx^®^ test. Identical conditions are required for patients with advanced epithelial ovarian cancer to be eligible for treatment with Olaparib [73] and rucaparib [20], metastatic pancreatic cancer patients for maintenance treatment with olaparib [74], and metastatic castration-resistant prostate cancer (mCRPC) patients for olaparib [75] and rucaparib [76] treatments [70]. A negative result can potentially hide a somatic *BRCA* mutation which is also an eligibility criterion for treatment in certain indications [72].

FoundationOne^®^ CDx test, developed by Foundation Medicine, is the second commercial test that has been recommended by ASCO and approved by the FDA as an HRD companion diagnostic for PARPi treatment in mCRPC and ovarian cancer. Using DNA extracted from FFPE, this test is based on hybrid-capture NGS and allows the detection of substitutions, indels, and CNAs in 324 genes, but also gLOH, and microsatellite instability (MSI) and tumor mutational burden (TMB) status. In addition, determination of HRD status is achievable with the FoundationOne^®^ CDx test thanks to *BRCA* status and gLOH. Thus, tumors are considered HRD if *BRCA* status is mutated or the gLOH ≥ 16% (LOH high). It is important to note that some large-scale deletions and rearrangements in *BRCA1/2* may not be detected, inducing false-negative results in approximately 5% of ovarian cancer patients and that samples must contain a minimum of 35% of tumor cells for conclusive gLOH [77]. FoundationOne^®^ CDx identifies prostate cancer patients as eligible for olaparib if a deleterious or suspected deleterious germline or somatic mutation in HRR genes (*ATM*, *BARD1*, *BRCA1/2*, *BRIP1*, *CDK12*, *CHEK1/2*, *FANCL*, *PALB2*, *RAD51B/C/D*, and *RAD54L*) is found [75]. For advanced ovarian cancer patients, only germline or somatic *BRCA* status is required to identify eligible patients for olaparib as maintenance treatment [78], and rucaparib in relapsed ovarian cancers [79].

Based on the same technology, FoundationOne^®^ Liquid CDx is another test provided by Foundation Medicine and approved by the FDA. Contrary to FoundationOne^®^ CDx, FoundationOne^®^ Liquid CDx requires circulating cell-free DNA (cfDNA) extracted from plasma derived from anti-coagulated whole blood. Substitutions and indels are detected in 311 genes, rearrangements in 8 genes (*ALK*, *BRCA1/2*, *NTRK1/2/3*), and CNA in 3 genes (*BRCA1/2* and *ERBB2*). Variant allelic frequencies (VAF) of *BRCA1*, *BRCA2*, and *ATM* rearrangements must be ≥ 0,25% and ≥ 0,85% (or ≥ 0,11% and ≥ 0,15% if short variants) to ensure olaparib and rucaparib efficiencies, respectively [80]. In fact, FoundationOne^®^ Liquid CDx is approved to identify mCRPC eligible for olaparib if an alteration in *ATM*, *BRCA1* and/or *BRCA2* is found in cfDNA [81], and for rucaparib if a *BRCA1* or *BRCA2* mutation in cfDNA is detected with the test [82]. For advanced ovarian cancer, FoundationOne^®^ Liquid CDx allows to detect deleterious *BRCA1/2* mutations, which make patients eligible for rucaparib [83].

Regarding commercial tests without FDA approval, the Tempus HRD test provided by Tempus Labs is an add-on test available after DNA analysis by the Tempus xT test. This NGS-based method consists of a hybridization capture panel of 648 genes including 18 HRR genes (*ATM/R/RX*, *BARD1*, *BRCA1/2*, *BRIP1*, *CDK12*, *CHEK2*, *FANCA*, *HDAC2*, *MRE11*, *NBN*, *PALB2*, *RAD51B/C/D*, and *RAD54L*) and covering 3.6 Mb of the human genome. A minimum of 40% tumor purity is required. To predict the HRD status of FFPE samples, gLOH, LOH at specific *BRCA1/2* loci, and deleterious *BRCA1/2* mutations are determined. Tumor is considered HRD (or “*BRCA*-biallelic”) if a homozygous deletion, a pathogenic germline/somatic mutation with overlapping LOH of the other allele or presence of both germline and somatic mutation of BRCA1/2 is found. For breast and ovarian cancers, the gLOH threshold is set at 21% and 17%, respectively. For other cancer types, Tempus xR, a whole-transcriptome RNA sequencing panel, is more accurate for HRD status determination. This HRD-RNA model is a logistic regression model. The HRD-RNA score is comprised between 0 and 100, a score ≥ 50 predicting an HRD tumor. A third category called “HRD-ambiguous” gathers tumors with a *BRCA1/2* monoallelic loss or reversion mutations, or any mutations in *CDK12*, *PALB2* or *RAD51B/C/D*. In contrast to the HRD-DNA model, the HRD-RNA model discriminates samples with *BRCA1/2* reversion mutations and also provides a dynamic HRD phenotype. A positive correlation is found between HRD status prediction obtained from the Tempux xT HRD test and the CHORD classifier. However, discrepancies are reported: a lower HRD rate is obtained for pancreatic cancer with Tempus compared to CHORD and a higher rate for metastatic mesothelioma and sarcoma. Nevertheless, HRD status has not yet been approved as a biomarker for PARPi in these indications [84].

HRD status can also be determined by CancerPrecision^®^ from CeGaT. This test requires FFPE tumor block or tissue slides with a minimal of 20% tumor content or blood for liquid biopsy, and EDTA blood for comparison with the corresponding normal sample. Over 700 genes are covered by this panel. The HRD score is calculated from HRD-LOH, LST, and TAI signatures. Evidence of HRD is reported if the HRD score is ≥ 30. In addition, pathogenic variants of *BRCA* are described. A molecular tumor board (MTB) is included in the sample report in order to suggest treatment options based on the results obtained [85].

Caris Life Sciences offers another commercial test named Molecular Intelligence^®^ Comprehensive Tumor Profiling that only requires tumor material to be sent (FFPE block or unstained slides). The Molecular Intelligence (MI) Exome™ NGS-based assay is used to provide HRD status using DNA isolated from FPPE. Indeed, 250,000 SNPs are analyzed to obtain the score of genomic instability of the tumor. At first MI Exome™ was a designed panel with 592 target genes (including *ATM*, *BARD1*, *BRCA1/2*, *BRIP1*, *CDK12*, *CHEK1/2*, *FANCL*, *H2AX*, *PALB2*, *RAD51B/C/D*, and *RAD54L*), and now, around 22,000 genes are enriched in the panel (analogous to WES). A minimum of 20% tumor purity is required. With this panel, an HRD status is reported as positive if a pathogenic or likely pathogenic variant in *BRCA1* and/or *BRCA2* is found or if the sum of gLOH and LST is high. The gLOH assay threshold is ≥ 16%. To note, the Caris HRD Status serves as a biomarker only for niraparib, olaparib, and rucaparib indications in ovarian cancers, and is not available in all locations [86].

Different panels are commercially available to assess *BRCA* and other HRR genes mutational status (Table 1). As with the tests listed above, no handling is required, the samples need to be shipped. However, the HRD score cannot be obtained with these panels.

**Table 1:** Panels commercially available for the detection of *BRCA* and homologous recombination repair (HRR) genes status and not applicable in lab*

Provider	Name of panel	Number of target genes	Sample type	Genes assessed	Time to get results
AmbryGenetics	BRCANext^®^ [87]	18	Blood, saliva	g*BRCA* and HRR genes	14–21 days
	BRCANext-*Expanded*^®^ [88]	23			
	CancerNext^®^ [89]	34			
	CancerNext-Expanded^®^ [90]	71			
	BRCAPlus^®^ [91]	13		g*BRCA*	7–10 days
Centogene [92]	CentoBreast^®^	28	Blood, saliva	g*BRCA* and HRR genes	15 days
	CentoCancer^®^	67			
	CentoCancer^®^ comprehensive	115			
GeneDX	Comprehensive Common Cancer Panel [93]	47	Blood, saliva	g*BRCA* and HRR genes	14 days
	Common Cancer Management Panel [94]	38			
Guardant Health	Guardant360^®^ CDx [95]	55	Plasma	*BRCA* and 4 HRR genes	7 days
	Guardant360 TissueNext™ [96]	84	FFPE	*BRCA* and HRR genes	14–21 days
	Guardant360^®^ [97]	83	Plasma	*BRCA* and HRR genes	10 days
Invitae [98]	BRCA1 and BRCA2 STAT Panel	2	Blood, saliva	g*BRCA*	5–12 days
	Multi-cancer Panel	70	Blood, saliva	g*BRCA* and HRR genes	10–21 days
	Common Hereditary Cancers Panel	48			
	Hereditary Breast and Gyn Cancers Guidelines-Based Panel	19			
NeoGenomics	NeoTYPE^®^ HRR Profile [99]	30 + PD-L1 by IHC	FFPE tissue	*BRCA* and HRR *genes*	14 days
University of Washington Medical Center	BROCA Cancer Risk Panel [100]	74	Blood, saliva	g*BRCA* and HRR genes	14–28 days

* Current as of March, 2024. IHC, immunohistochemistry

Compared to centralized commercial HRD assays, the commercial in-house tests allow for independent testing at the local level. Samples preparation and analyses are therefore performed in the same laboratory. Results are also obtained with a shorter period time with commercial in-house test compared to Myriad Genetics or Foundation Medicine tests (7 days vs. 18 days) [101]. Nevertheless, these independent tests are not approved by the FDA for determining the HR status.

The AmoyDx^®^ HRD Focus panel is one of the commercial in-house tests for HRD status, available at Amoy Diagnostics. This in vitro diagnostic assay uses DNA isolated from FPPE, which is then sequenced on a NextSeq 550 system using a hybridization capture panel covering 1.5 Mb of the human genome (including all exons of *BRCA1* and *BRCA2*) and targeting 24,000 SNPs. The AmoyDx^®^ HRD Focus assay (CE-IVD) enables to estimate the Genomic Scar (GS) Score (GSS), validated in 2022 by Yuan et al. The GS model, a machine learning method, is used to predict HRD events. Indeed, the GSS is based on chromosomal CNVs and consists of the sum of weight of 3 CNV categories: length of CN (LCN) (large if > 15 Mb, middle for 10–15 Mb, and small if < 15 Mb), site of CN (SCN) (centromere, telomere, and other sequences), and type of CN (TCN) (LOH, and allele-specific/balance CNV). HRD status is considered as positive for a GSS ≥ 50 and/or a pathogenic/likely pathogenic *BRCA1/2* mutation [102]. There is a high concordance with the MyChoice^®^ CDx test (87.8%) [101].

ThermoFisher Scientific provides three panels for the detection of HRD: the Ion Torrent™ Oncomine™ BRCA Research Assay, the Ion Torrent™ Oncomine™ HRR Pathway Predesigned Panel, and the Ion Torrent™ Oncomine™ Comprehensive Assay Plus (OCA+). The first two panels target 2 and 28 HRR genes, respectively, and are able to determine the *BRCA* status. HRD-LOH score, TAI, and LST can only be obtain with OCA+, which is a 501 genes panel covering 1.4 Mb of the human genome. OCA + requires 20 ng of FFPE isolated DNA, and prepared libraries must be sequenced with ThermoFisher Scientific sequencing instrument. A genomic instability metric (GIM) value is calculated to determine the HRD status, with a threshold ≥ 16. GIM scores obtained with OCA + are concordant with GIS scores obtained with the MyChoice^®^ CDx test [103].

Another commercial in-house test is the TruSight™ Oncology (TSO) 500 HRD kit supplied by Illumina (not available in Japan). TSO 500 HRD is a panel of 523 genes covering 1.94 Mb of DNA. A 40 ng of DNA input (isolated from FFPE sample) allows SNVs, indels, CNVs, and splice variants, including *BRCA* large rearrangements, to be called. A minimum of 32% tumor content is required [104]. HRD-LOH, LST, and TAI are assessed, resulting in a GIS with 100% specificity. GIS reported by the TSO 500 test is highly concordant with GIS obtained by the MyChoice^®^CDx test. The overall agreement between the two tests (including HRD GIS, *BRCA* status and HRD status) is > 93% [105].

As observed with centralized commercial testing, some commercial in-house tests are offered by different manufacturers, but they only provide information on the status of *BRCA* and other HRR genes. The SureSelect Cancer CGP Assay [106] and the SureSelect Cancer All-IN-One Solid Tumor Assay [107] are provided by Agilent and targeted 679 and 20 genes, respectively. Illumina offers two panels of 2 and 161 genes, the AmpliSeq™ BRCA and AmpliSeq™ Comprehensive panels, respectively [108]. Additionally, Qiagen provides four panels for assessing *BRCA* and other HRR genes, including AmpliSeq™ and QIAseq panels, which can be sequenced on either Illumina or ThermoFisher sequencing instrument technologies [109].

Custom panels represent alternative methods to commercial panels. Customized libraries can be designed from a commercial panel. A bioinformatics data analysis pipeline can be implemented using either in-house or custom scripts, and HRD scores are calculated using this pipeline. Custom panels are deployed in decentralized academic laboratories. Standardization of these HRD assays in academic laboratories is mandatory to provide reliable alternative options to commercial HRD tests. The aim is generally to obtain HRD assays that are cheaper, faster, and have a lower failure rate [41, 110]. Capoluongo et al. compared an academic genomic assay using a custom panel with the MyChoice^®^ CDx test. Samples used came from patients enrolled in the MITO16A-ManGO-OV2 trial (NCT01706120). Concordance between the two tests was good, demonstrating the feasibility of this academic assay to determine HRD status [41]. Denkert et al. successfully performed a custom panel developed with Myriad Genetics in a decentralized laboratory. Compared to the MyChoice^®^ CDx test, the concordance for HRD status between both tests was 97.1% [110], rendering HRD testing in academic laboratories promising.

The Leuven HRD test was introduced in 2022 by Loverix et al. This is an hybridization capture panel developed using the PAOLA-1/ENGOT-ov25 cohort (NCT02477644) to identify patients with HRD advanced ovarian cancer (AOC) eligible for Olaparib [111]. Around 90,000 genome-wide SNPs are included in one panel, and coding exons of 9 genes (*BARD1*, *BLM*, *BRCA1/2*, *BRIP1*, *PALB2*, *RAD51C/D*, and *TP53*) are covered with a second panel. DNA extracted from FFPE tissue was used for this assay and the sequencing was performed using a NovaSeq 6000 sequencer (Illumina). HRD status was determined by measuring GIS (LOH in combination with TAI and LST) with Allele-Specific Copy number Analysis of Tumor (ASCAT), and/or a pathogenic or likely pathogenic mutation of BRCA. Finally, a tumor was considered HRD positive if the GIS was ≥ 56 and/or a pathogenic/likely pathogenic mutation of BRCA1/2 was found. The threshold was set at 56 following analyses of the CLIO/BGOG-ov10 cohort (NCT02822157). The results obtained by the Leuven HRD test are correlated with results obtained by the MyChoice^®^ CDx test [112].

The NOGGO GIS v1 Assay was presented in 2023 by the Northeastern German Society for Gynecologic Oncology (NOGGO). This solution is based on the Agilent XT HS2 and SNPs backbone using a hybridization capture panel that covers 57 genes (including *BRCA1* and *BRCA2* genes) and targets 20,000 SNPs. The GIS (called “NOGGO GIS”) is estimated using the OneSeq CNV Backbone (Agilent Technologies). To perform the test, 50 ng of extracted FPPE samples with a minimum of 30% of tumor content are required. Overall, the NOGGO GIS v1 Assay can be easily implemented in laboratories with the resources to set up customized NGS workflows. HRD status is considered positive if the NOGGO GIS is ≥ 83 and/or a pathological *BRCA1* or *BRCA2* mutation is found. The NOGGO GIS Assay identifies reliably HRD patients included in the PAOLA-1/ENGOT-ov25 trial and is comparable to the MyChoice^®^ assay [113].

Since the 2020s, different HRD score algorithms have been developed to improve HRD assessment using NGS data generated from targeted gene panels. MUTation AnaLysIS toolKit (Mutalisk) [114] and Signature Multivariate Analysis (SigMA) [115] algorithms provide a comprehensive analysis of HRD-related mutational signatures using panel. R packages are available for all tools, making them easy to use. In contrast, HRD-Multi-Instance Learning (HRD-MILN), a machine learning-based approach, predicts HRD status from HRD-LOH signatures and panels. Some bioinformatics tools detect the HRD status using the HRD-LOH, TAI and LST signatures, as with Genomic Instability Scar (GIScar) [116] (a Software As A Service (SAAS) solution) and Genomic Scar Analysis (GSA) [117]. The HRD_CNA_ [118] is a gradient boosting machine (GBM) which predict the HRD status *via* 10 CNA features.

#### NGS data obtained by shallow WGS (sWGS)

Shallow WGS (sWGS) or Low Coverage (LC) WGS is another sequencing method consisting of low-pass WGS. sWGS is a less expensive alternative to WGS. In sWGS, coverage depth and sequencing capacity are reduced, but CNA detection is highly accurate and coverage breadth is greater than that of WGS [119]. However, despite the good correlation between HRD scores obtained from sWGS and WGS data, the low cellularity of certain tumors and the presence of GC bias have been shown to impact sWGS results [120]. Indeed, samples with tumor cell content below 30 pose challenges for CNVs calling *via* bioinformatics tools designed to determine HRD status from sWGS data. Its phenomenon can be explained by the dilution of DNA from tumor cells in DNA from non-tumor cells, which reduces the tumor-specific signal and tends difficult to distinguish CNVs from background noise [121]. The presence of subclonal heterogeneity in these low-purity samples is a second concern, resulting in dilution of the CNVs signal across multiple populations [122]. The risk of false negatives is consequently elevated for this type of sample [123]. This underscores the necessity for the use of specific tools in this context.

HRD status can be determined with the R package shallowHRD. A sWGS with around 1X coverage of DNA extracted from FFPE samples is a prerequisite for using this software tool. Large-scale genomic alterations (LGAs) (the number of CN breakpoints) are given, and HRD status is positive if LGAs > 20 (“specific” cut-off). Between 15 and 20 LGAs, cases are considered borderline, and under 15 LGAs (“sensitive” cut-off), patients are HRP [124]. ShallowHRDv2, developed at Institut Curie (France), is highly concordant with MyChoice^®^ CDx (91%), and the number of non-contributory analyses is lower with shallowHRDv2 compared to MyChoice^®^ CDx [125, 126]. Moreover, the shallowHRD method can be performed after the determination of *BRCA* mutation and methylation status with another method, in order to facilitate identification of HRD status and reduce cost [127].

Another bioinformatics tool based on sWGS data is ChosenHRDw, developed in 2022 by Zhang et al. The HRD status is predicted using 8 HRD-related signatures in a random forest model including chromosomal instability index (CIN index), CNV ratio, HRD-LOH, LST and TAI, HRR index (index of HRR gene instability), CN ratio in transcription factor binding sites (TFBS), and weighted genome instability index (wGII). This tool is highly concordant with HRDetect (AUC 0.93), but further experiments are required, including analysis of a larger cohort [128].

The AcornHRD algorithm can also be used to obtain HRD status. An estimation of large-scale copy number alteration (LCNA) events is performed to calculate the HRD score, with a threshold value of 10. The results obtained by Pan et al. indicated that the AcornHRD tool outperformed the shallowHRD method in determining HRD status. However, a comparison with the FDA-approved HRD tests is needed to confirm the accuracy of the AcornHRD algorithm [129, 130].

Moreover, two commercial tests are locally available to assess HRD status through the analysis of sWGS data without material shipping: the SeqOne HRD Solution and the SOPHiA DDM™ HRD Solution.

The SeqOne HRD solution is available on the SeqOne Genomics testing plaform. This platform analyzes data obtained using validated techniques (e.g. Agilent Magnis or Bravo for NGS library preparation, and Illumina NextSeq 550 or NovaSeq 6000 as sequencing technology), making it easy to implement in laboratories. Determination of HRD status is based on two testing approaches. The first strategy uses a 16 genes panel with coverage of at least 35X to obtain *BRCA1/2* mutations status, combined with a sWGS with coverage of at least 0.3X to obtain the SeqOne HRD Score. This score is determined using two features of GI (number of LGAs and loss of parental copy (LPC)) and two features of CNV (amplification of *CCNE1* and *RAD51B*). The second strategy is composed of two steps: a gene panel is performed to assess *BRCA* status, and if the tumor is wt*BRCA*, a secondary sWGS is conducted to determine HRD status *via* the SeqOne HRD scores. DNA isolated from FFPE is used with a minimum cell purity of 20%. A SeqOne HRD status is positive if a pathogenic mutation of *BRCA1* or BRCA2 is found and/or the SeqOne score is higher than 50% [131]. Compared to the MyChoice^®^ CDx test, the SeqOne HRD solution similarly selects ovarian cancer patients for olaparib *plus* bevacizumab treatment (PAOLA-1 trial) and both are concordant (95%) [132, 133].

The SOPHiA DDM™ HRD Solution is provided by SOPHiA Genetics™. In this solution, a targeted capture panel of 28 HRR genes (*AKT1*, *ATM*, *BARD1*, *BRCA1/2*, *BRIP1*, *CCNE1*, *CDK12*, *CHEK1/2*, *ESR1*, *FANCA/L/D2*, *FGFR1/2/3*, *MRE11*, *NBN*, *PALB2*, *PIK3CA*, *PPP2R2A*, *PTEN*, *RAD51B/C/D*, *RAD54L*, *TP53*) is combined to a sWGS at 1X of depth [134]. This test requires 50ng of DNA isolated from FFPE sample and a minimal cell purity of 30%. The MUSKAT™ algorithm identifies gene amplifications, while the PEPPER™ algorithm identifies indels and SNVs. The *BRCA* status is associated with the Genomic Integrity Index (GII) to determine the HRD status, providing information about the causes and consequences of HRD, respectively. This GII score is obtained from sWGS data using the deep learning algorithm GIInger™. All these algorithms are available on the SOPHiA DDM™ Platform. A SOPHIA DDM HRD status is positive if the GII is ≥ 0 [135, 136]. The MyChoice^®^ CDx test and the SOPHiA DDM™ HRD solution have a high level of agreement, with a concordance rate of 94.5% [137].

#### NGS data obtained by WGS and WES

Compared to the original SNP array technique, NGS methods are just as effective at detecting HRD tumors [120]. Among them, the WGS is the most appropriate method for detecting HRD mutational signatures. All genetic material, including coding and non-coding regions, is sequenced in this complete method [138, 139]. However, WGS is an expensive technique and difficult to implement in clinical routine. WGS data can detect large scale rearrangements, but the large amount of data makes analysis difficult. In contrast, the WES method offers an attractive alternative to WGS by sequencing only coding regions, making it easier to use [120, 139].

In 2017, Davies et al. developed an HRD classifier based on WGS data: the HRDetect. This lasso logistic regression model is established on six distinguishing mutational signatures including the microhomology-mediated indels at the breakpoint junctions, the HRD index score (based on SNP array data), the RS3/5, and the SB3/8. The HRDetect score can identify tumors with a probability of *BRCA1/2* deficiency. This algorithm classifies as HRD a tumor with biallelic *BRCA1/2* inactivation prediction with a 98.7% sensitivity (AUC 0.98), despite the constraints related to the use of FFPE samples for WGS analyses (low tumor cellularity, or noise from formalin fixation). The threshold was set at 70% following analyses of breast cancer data and tumors scoring below 70% are considered as HRP [27]. In addition to breast cancer, HRDetect is validated for ovarian and pancreatic cancers. However, for ovarian cancers, a 99% cut-off instead of 70% seems to offer better accuracy (0.95 vs. 0.83) [140].

The Classifier of Homologous Recombination Deficiency (CHORD) is a second HRD classifier, developed in 2020 by Nguyen et al. Using WGS data, CHORD is based on 3 categories of 29 mutational features, including indels (characterized by the presence or absence of sequence homology and tandem repeats), SNVs classified by base substitution type, and SV classified by length and type. CHORD was validated on a pan-cancer cohort (comprising 20 cancer types) and a threshold was arbitrarily set at 0.5. CHORD can distinguish *BRCA1*-type HRD samples from *BRCA2*-type HRD samples through 1-100 kb structural duplications [141]. The accuracy of detection *BRCA1/2* deficiency in ovarian cancers by CHORD training can be increased with a 0.84 cut-off instead of 0.5 (0.97 vs. 0.91). In contrast, a lower threshold for breast and pancreatic cancers (0.1 and 0.13, respectively) resulted in the same level of accuracy [140]. To note, CHORD cannot determine HRD status on MSI samples [141].

Similar to NGS data derived from targeted gene panels, NGS data obtained through WGS or WES can be utilized by various HRD scoring algorithms to enhance the determination of HRD status. The three R packages Mutalisk [114], SigMA [115] and Yet Another Package for Signature Analysis (YAPSA) [142] provide insight into HRD status through the analysis of mutational signatures. Conversely, others tools employ genomic scars (HRD-LOH, TAI and LST signatures) to predict this status by analyzing NGS data generated from WES and WGS, such as ASGAD [143], ovaHRDscar [144], or scarHRD [61]. In the context of HRD_CNA_ [118], 10 CNA features are employed to predict the HRD status. The use of algorithms depends on the type of sequencing data and information available.

### Determination of HRD status using optical genome mapping (OGM)

More recently, Optical Genome Mapping (OGM) is an alternative technology to SNP arrays and NGS methods for identifying patients with HRD. Indeed, this non-sequencing-based method can detect large SVs such as CNVs, indels > 5 kbp, inversion > 70 kbp, and translocations > 70 kpb, at VAF of 5%, and can determine HRD score of samples through the sum of HRD-LOH, LST and TAI signatures [145]. Ultra-high molecular weight (UHMW) DNA from fresh or frozen tissue or blood are required for this assay. Labelled DNA is then transferred into a chip. The Saphyr^®^ system is an example of OGM system provided by Bionano™ genomics [146] and HRD score are calculated using the NxClinical software from OGM data [147]. Determination of HRD status seems to be more sensitive with OGM method than with NGS method but further experiments are needed to evaluate this technology for HRD status determination [148].

## Discussion

PARPi are most effective in patients with *BRCA1* or *BRCA2* loss-of-function mutations, followed by those with an HRD phenotype. Identification of the BRCAness population is thus crucial, despite the many barriers to HR status assessment [149]. In fact, there are other ways that confer sensitivity to PARPi, irrespective of *BRCA* status, but not all the mechanisms leading to the HRD phenotype are yet known. Although it can be now confirmed that inactivation of *PALB2* and *RAD51* paralogs may explain the HRD phenotype [150], there is currently insufficient evidence to fully demonstrate the involvement of additional HRR genes in this phenotype. For example, *CCNE1* amplification/gain, which is a predictive biomarker of PARPi and platinum-based treatment resistance in epithelial ovarian cancers [151], is mutually exclusive with a pathogenic *BRCA1/2* mutation in ovarian cancers [152]. However, a co-occurrence of a BRCAness phenotype and an amplification of *CCNE1* remains a possibility. Nonetheless, the overall survival of these BRCAness patients exhibiting an HRD phenotype and *CCNE1* amplification/gain is not improved when treated with PARPi or platinum-based chemotherapy, in contrast to BRCAness patients without *CCNE1* amplification/gain. This suggests that *CCNE1* amplification/gain has a greater influence on response to these treatments compared to the HRD phenotype in wt*BRCA* patients [153]. As a result, a comprehensive panel including various HR-related genes is commonly used in commercial or academic tests to identify HRD patients. The issue of determining HRD status using either single or multiple mutated genes in the HR pathway is also a concern. Furthermore, alterations outside of the HRR pathway may be associated with an HRD phenotype, although not solely accountable for it. As an illustration, loss of *TP53* may lead to a higher gLOH score, particularly when coupled with loss of *PTEN*. In contrast, the MSI status exhibits an inverse correlation with the manifestation of the HRD phenotype [154]. Consequently, all these features must be considered when studying the HRD status of tumors.

The HRD phenotype is dynamic and subject to change over time. Highlighting a positive or negative HRD status is mainly obtained with genomic scars, but it may not necessarily be indicative of the HRD status at the time of treatment. Phenomena such as *BRCA* reversion mutations or acquisition of an HRP phenotype after biopsy cannot be detected through the sole analysis of genomic scars. As they reflect genomic history rather than ongoing processes, using these markers to obtain HRD status poses a risk of false positive results. Functional tests are thus considered the optimal method as they provide real-time information on HRD status and have a good predictive value for response to treatment with PARPi or platinum-based chemotherapy. However, this method requires a substantial quantity of fresh tissue, which makes it impractical for routine clinical practice, despite ongoing efforts to implement this technique on FFPE samples [155, 156].

Currently, the recommended approach for determining whether HRD status of a patient is positive or negative in clinical practice is through indirect determination. It relies on the identification of mutations in HR-related genes, mutational signatures, or genomic scars, either individually or in combination. The primary tests used to determine HRD status are depicted in Table 2, along with their clinical validation in specific tumor types.

**Table 2:** Summary table of available tests for homologous recombination deficiency (HRD) status (non-exhaustive list)*

Test	Type of data	HRD status determination ^i^	PPA (%)	NPA (%)	OPA with myChoice^®^ CDx (%)	Clinical validation	Cut-off for HRD status determination	Ref.
Centralized commercial tests								
CancerPrecision^®^	Panel	HRD score (LOH, LST, TAI)	NA	NA	NA	No	60	[85]
FoundationOne^®^ CDx (FDA approved)	Panel	LOH score	67.6	85.7	77	Yes (OC, PC)	16%	[165]
Molecular Intelligence^®^	Panel	Caris GSS (LOH, LST)	96	99	98.4	Yes (OC)	42	[166]
MyChoice^®^ CDx (FDA approved)	Panel	GIS (LOH, TAI, LST)	Reference	Reference	Reference	Yes (BC, OC)	42	[101]
Tempus xT HRD Test	WES	LOH	NA	NA	NA	Yes (BC, OC)	21% (BC), 17% (OC)	[84]
Tempus xR HRD test	WTS	F1-score	NA	NA	NA	Yes (pan-cancer)	50	[84]
Commercial in-house tests								
AmoyDx^®^ HRD Focus panel	Panel	GSS (LOH, LST, TAI)	75	100	88.6	Yes (OC)	50	[167]
Oncomine™ Comprehensive Assay Plus	Panel	GIM (LOH, TAI, LST)	98.1	79.4	90.7	Yes (OC)	16%	[168]
OncoScan™ CNV	WG CNV	GIS (LOH, TAI, LST)	88.9	68.2	77.5	Yes (OC)	42 (with Rediscore v2.0) or 15 (with OncoscanR)	[167]
SeqOne HRD	sWGS, panel	SeqOne HRD score (LGA + LPC + CNV)	95.3	95.1	95.3	Yes (OC)	50%	[133]
SOPHiA DDM™ HRD Solution	sWGS, panel	GII (LOH, TAI, LST)	89.4	96.6	93	Yes (OC)	0	[169]
TruSight™ Oncology 500 HRD	Panel	GIS (LOH, TAI, LST)	95.2	96.8	> 93	Yes (BC, OC)	42	[170]
Academic tests								
Leuven HRD	Panel	GIS (LOH)	95	86	91	Yes (OC)	56	[112]
NOGGO GIS v1 Assay	Panel	GIS (LOH, TAI, LST)	94.6	87	90.4	Yes (OC)	83	[113]
Bioinformatics tools								
ChosenHRDw	sWGS	HRD score, CIN index, wGII, CNV ratio, HRR index, TFBS ratio	87	90	NA (93% with HRDetect)	No	30	[128]
CHORD	WGS	Sig3, Sig5	NA	NA	NA (99% with HRDetect)	Yes (pan-cancer)	0.5	[141]
HRDetect	WGS	Sig3, Sig5	> 95	> 95	NA	Yes (BC, OC, PC)	70%	[27]
shallowHRDv2	sWGS	LGA	95.1	92	93.7	Yes (BC, OC, PrC)	20	[126]
SigMA	WES, WGS, panel	Sig3	> 65	90	NA	Yes (pan-cancer)	Sig3 positive	[163]

* Current as of March, 2024

^i^ in addition to *BRCA* status determination

BC, Breast Cancer; CIN index, chromosomal instability index; CNV ratio, copy number variations ratio; FDA, Food and Drug Administration; GIS, genomic instability score; HRR, homologous recombination repair; LCNA, large-scale copy number alteration; LGA, Large-scale Genomic Alteration; LOH, loss of heterozygosity; LST, large-scale transition; NA, not assessed; NPA, negative percent agreement; OC, Ovarian Cancer; OPA, overall percent agreement; PC, Pancreatic Cancer; PPA, positive percent agreement; PrC, Prostate Cancer; Sig3/5, COSMIC Signature 3/5; sWGS, shallow whole genome sequencing; TAI, telomeric allelic imbalance; TFBS, transcription factor binding sites; WES, whole exome sequencing; wGII, weighted genome instability index; WGS, whole genome sequencing; WTS, whole transcriptome sequencing

Sequencing methods seem the most appropriate approaches for identifying genomic mutations. To pinpoint HRD patients, utilizing a custom panel limited to a few genes seems to be one of the most efficient clinical approaches. This sequencing method is the most cost-effective and provides the quickest outcomes. Nevertheless, this strategy is not suitable for detecting mutations in all HRR genes. While WGS and WES can overcome these limitations, their widespread use is hindered by high costs, the need for strong bioinformatics expertise, and the complexity of interpreting mutations. Indeed, beyond the well-described *BRCA* mutations found in databases such as BRCA Exchange (https://brcaexchange.org/), interpreting variants of other HR-related genes poses a challenge, in addition to knowing which HR-related genes to take into consideration. When mutations are not described, their interpretation depends on the validator. This is particularly true for variants of unknown significance (VUS). Yoshida et al. proposed a model for VUS reclassification of BRCAness genes, with the aim of improving the assessment of these variants [157]. Nevertheless, because nonsense mutations, frameshifts and splicing mutations are more likely to alter proteins structure than other types of mutations, it is generally accepted that they are pathogenic [158].

For now, the gLOH, LST and TAI signatures, combined in the GIS (or HRD score or HRD-sum), are the most accurate features that give an overview of genomic instability which may be the result of a deficiency in the HR pathway. A value above the threshold designates a positive HRD status, while a value below the defined threshold denotes a negative HRD status and classifies the patient as HRP. The HRD score is thus a dichotomous variable that tends to be the clinical gold standard for determining patients’ HRD status, but some aspects still require clarification. Despite concerted efforts to establish a universal clinical threshold for assessing pan-cancer HRD status, it would seem impossible to set up a consortium. For example, the GIS threshold used to identify ovarian tumors which are HRD positive is similar to that employed to determine the HRD status of triple-negative breast cancers but not for estrogen receptor-positive breast cancers [159]. In the pan-cancer study of Sokol et al., the gLOH threshold was established between 14 and 16% for numerous cancers. However, for breast and prostate cancer, the cut-off was set over 16% and less than 14%, respectively [160]. As both of these cancers can be treated with PARPi and platinum salts, the use of a universal gLOH threshold may exclude some patients who could benefit from the treatment. This raises the question of whether the threshold value is continuous rather than discontinuous, requiring adaptation to each tumor subtype as well as to the treatment used.

Deficiency in HR pathway may also be assessed *via* algorithms that utilize mutational signatures for HRD status assessment. Notably, the SBS3 signature, which is typically linked to HRD, alone can overestimate the count of HRD patients [161]. In line with genomic scars, a combination of multiple mutational signatures results in better accuracy for HRD status assessment than the use of a single signature [162], as demonstrated by tools such as HRDetect and CHORD. Nonetheless, thresholds specific to each tumor subtype must still be applied, and it seems necessary to determine MMR status before using these classifiers to avoid false-negative results [140]. Moreover, these tools require WGS data, which is not yet the standard in routine clinical practice. In this regard, the algorithm SigMA appears to be more adapted, as they utilize NGS data obtained with specific panels [163].

In addition to mutational signatures, bioinformatics tools can identify genomic scars using targeted panel sequencing data, such as GIScar [116], which is more applicable in routine clinical practice. The shallowHRD tool, which is based on the study of sWGS data, seems to be an accurate algorithm for identifying HRD status, while being a well-suited tool for a routine clinical use. Its application in breast, pancreatic, and prostate cancers suggests a potential pan-cancer approach, making sWGS an interesting alternative method [164].

Once the concern of adapting threshold according to tumor type resolved, the challenge of situations in which the score is close to the cutoff persists. Moreover, it is often in such predicaments that disagreements arise between techniques [171]. Because decisions whether assigning a patient in the HRD or HRP group will have therapeutic consequences, using multiple sources to obtain HRD status makes sense, i.e. a combination of information about causes and consequences of HRD [172]. The only two HRD tests approved by the FDA are offered by Myriad Genetics and Foundation Medicine. Most of the other available methods that determine HRD status compared their results with those obtained with the MyChoice^®^ CDx test. However, these centralized approaches have disadvantages, such as slow response time and a high rate of sample rejection. Indeed, compliance criteria are sometimes impossible to meet due to the disparity of clinical samples. In response to changing circumstances, in-house testing has grown in popularity. Nevertheless, these decentralized tests are not yet optimal as there is no universal consensus on which genes to include in panels or on the cut-off value of HRD scores depending on the type of tumor. The key to ensure accurate results is to harmonize these characteristics. Commercial NGS tests such as those provided by Amoy Diagnostics or SOPHiA Genetics seem be to the better alternative to the centralized tests. Theses commercial assays can accurately determine HRD status in the same laboratories where samples are prepared (sequencing capabilities permitting), with the added benefit that data analysis is performed directly by the proprietary software [101, 134]. In addition, most of them are highly concordant with each other, despite some discrepancies in test interpretation and final patient management, which is inherent in molecular testing when multiple parameters are considered [173].

The HRD phenotype is dynamic and subject to change over time. Highlighting a positive or negative HRD status is mainly performed with genomic scars obtained with FFPE samples, but it may not necessarily be indicative of the HRD status at the time of treatment. Phenomena such as *BRCA* reversion mutations or acquisition of an HRP phenotype after biopsy cannot be detected through the sole analysis of genomic scars. As they reflect genomic history rather than ongoing processes, using these markers to obtain HRD status poses a risk of false positive results. Functional tests are thus considered the optimal method as they provide real-time information on HRD status and have a good predictive value for response to treatment with PARPi or platinum-based chemotherapy. However, this method requires a substantial quantity of fresh tissue, which makes it impractical for routine clinical practice, despite ongoing efforts to implement this technique on FFPE samples [155, 156].

Sample quality is another limiting factor for the correct determination of HRD status. In fact, HRD score can be influenced by the tumor purity [174] and tumor fixation conditions, which can alter sequencing results due to the formalin presence [175]. To bypass non-contributory FFPE samples, HRD testing with cfDNA extracted from peritoneal fluid [176] or plasma is an excellent alternative method. The rapidity of sampling and the possible detection of *BRCA2* reversion mutations are the main advantages of liquid biopsy, allowing a longitudinal monitoring of HRD status [177]. With the rise of artificial intelligence in the current decade, research teams try to devise alternative methods for evaluating HRD status without the use of molecular biology. For instance, Lazard et al. developed a deep learning-based method allowing HRD status prediction by analyzing hematoxylin and eosin-stained (H&E) whole-slide images (WSIs). Although further experimentations are needed to clinically validate this solution, it highlights a cost-effective method that has the potential to be routinely used for the detection of phenotypes related to HRD [178]. However, these approaches also depend on the analysis of FFPE tissue, resulting in the same limitations as NGS analysis due to the tissue sampling bias associated with this method.

## Conclusions

Recent advances in sequencing and functional testing have made HRD status increasingly reliable. In addition to the Myriad Genetics MyChoice^®^ CDx reference test, there are now several other solutions available to obtain an accurate HRD status by combining multiple parameters. Nevertheless, further explorations are needed to identify which genes are specifically implicated in the HRD phenotype, with the aim to determine precisely what test for. Harmonizing the definition of the various HRD markers and their cut-off values will also be a key step in obtaining HRD status for patients.

## Data Availability

No datasets were generated or analysed during the current study.

## Abbreviations

ACNE: Allele-specific CN Estimation
AOC: Advanced Ovarian Cancer
ASCAT: Allele-Specific Copy number Analysis of Tumor
ASCO: American Society of Clinical Oncology
BER: Base Excision Repair
cfDNA: Cell-free DNA
CDx: Companion Diagnostic
CHIP: Clonal Hematopoiesis of Indeterminate Potential
CHORD: Classifier of Homologous Recombination Deficiency
CIN: Chromosomal INstability
CN: Copy Number
CNA: Copy Number Alteration
CNV: Copy Number Variation
DBS: Doublet-Base Substitution
DSBs: Double-Strand Breaks
FDA: Food and Drug Administration
FFPE: Formalin-Fixed Paraffin-Embedded
GI: Genomic Instability
GIM: Genomic Instability Metric
GIS: Genomic Instability Score
gLOH: Genomic Loss Of Heterozygosity
GS: Genomic Scar
GSS: Genomic Scar Score
HR: Homologous Recombination
HRD: Homologous Recombination Deficiency
HRP: Homologous Recombination Proficiency
HRR: Homologous Recombination Repair
ID: Small Insertion Deletion
IHC: ImmunoHistoChemistry
Indel: Insertion-Deletion
KEGG: Kyoto Encyclopedia of Genes and Genomes
LC: Low Coverage
LCN: Length of Copy Number
LGA: Large-scale Genomic Alterations
LPC: Loss of Parental Copy
LOH: Loss Of Heterozygosity
LST: Large-Scale Transition
mCRPC: Metastatic Castration-Resistant Prostate Cancer
MMR: MisMatch Repair
MRN: MRE11-RAD50-NBS1
MSI: MicroSatellite Instability
MTB: Molecular Tumor Board
NA: Not Assessed
NEHJ: Non-Homologous End Joining
NGS: Next-Generation Sequencing
NOGGO: Northeastern German Society for Gynecologic Oncology
NPA: Negative Percent Agreement
OCA: Oncomine Comprehensive Assay
OPA: Overall Percent Agreement
PARP: Poly(ADP-ribose) Polymerase
PARPi: Poly(ADP-ribose) Polymerase Inhibitor
PPA: Positive Percent Agreement
RPA: Replication Protein A
RS: Rearrangement Signature
SBS: Single-Base Substitution
SCN: Site of Copy Number
Sig: Signature
SNP: Single Nucleotide Polymorphism
SNV: Single Nucleotide Variation
SSBs: Single-Strand Breaks
sWGS: Shallow Whole Genome Sequencing
TAI: Telomeric Allelic Imbalance
TCN: Type of Copy Number
TFBS: Transcription Factor Binding Sites
TMB: Tumor Mutational Burden
TSO: TruSight™ Oncology
VAF: Variant Allele Frequency
WES: Whole Exome Sequencing
WG: Whole Genome
wGII: Weighted Genome Instability Index
WGS: Whole Genome Sequencing
wt: Wild-Type
WTS: Whole Transcriptome Sequencing
